# The mycobiome of a successful crayfish invader and its changes along the environmental gradient

**DOI:** 10.1186/s42523-023-00245-9

**Published:** 2023-04-11

**Authors:** Paula Dragičević, Ana Bielen, Jurica Žučko, Sandra Hudina

**Affiliations:** 1 Department of Biology, Faculty of Science, University of Zagreb, Horvatovac 102a, Zagreb, Croatia; 2 Faculty of Food Technology and Biotechnology, University of Zagreb, Pierottijeva 6, Zagreb, Croatia

**Keywords:** Invasive species, *Pacifastacus leniusculus*, ITS rRNA gene, Fungi, Microbiome

## Abstract

**Background:**

The microbiome plays an important role in biological invasions, since it affects various interactions between host and environment. However, most studies focus on the bacteriome, insufficiently addressing other components of the microbiome such as the mycobiome. Microbial fungi are among the most damaging pathogens in freshwater crayfish populations, colonizing and infecting both native and invasive crayfish species. Invading crayfish may transmit novel fungal species to native populations, but also, dispersal process and characteristics of the novel environment may affect the invaders’ mycobiome composition, directly and indirectly affecting their fitness and invasion success. This study analyzes the mycobiome of a successful invader in Europe, the signal crayfish, using the ITS rRNA amplicon sequencing approach. We explored the mycobiomes of crayfish samples (exoskeletal biofilm, hemolymph, hepatopancreas, intestine), compared them to environmental samples (water, sediment), and examined the differences in fungal diversity and abundance between upstream and downstream segments of the signal crayfish invasion range in the Korana River, Croatia.

**Results:**

A low number of ASVs (indicating low abundance and/or diversity of fungal taxa) was obtained in hemolymph and hepatopancreas samples. Thus, only exoskeleton, intestine, sediment and water samples were analyzed further. Significant differences were recorded between their mycobiomes, confirming their uniqueness. Generally, environmental mycobiomes showed higher diversity than crayfish-associated mycobiomes. The intestinal mycobiome showed significantly lower richness compared to other mycobiomes. Significant differences in the diversity of sediment and exoskeletal mycobiomes were recorded between different river segments (but not for water and intestinal mycobiomes). Together with the high observed portion of shared ASVs between sediment and exoskeleton, this indicates that the environment (i.e. sediment mycobiome) at least partly shapes the exoskeletal mycobiome of crayfish.

**Conclusion:**

This study presents the first data on crayfish-associated fungal communities across different tissues, which is valuable given the lack of studies on the crayfish mycobiome. We demonstrate significant differences in the crayfish exoskeletal mycobiome along the invasion range, suggesting that different local environmental conditions may shape the exoskeletal mycobiome during range expansion, while the mycobiome of the internal organ (intestine) remained more stable. Our results provide a basis for assessing how the mycobiome contributes to the overall health of the signal crayfish and its further invasion success.

**Supplementary Information:**

The online version contains supplementary material available at 10.1186/s42523-023-00245-9.

## Background

Introductions and successful spreading of invasive alien species (IAS) contribute to biodiversity loss, degrade the ecosystem structure and impair ecosystem services in the novel environment [1, 2]. Invasion success of each IAS is influenced by a variety of factors (e.g. [3, 4]), one of which is the interaction of microbial communities (i.e. the microbiome) with the invader during range expansion. Both the microbes in the novel environment and the microbes carried by IAS may play an important role in the process of invasion, since they affect host’s (i.e. invader’s) physiology, immune status, health and fitness [5–8]. Several hypotheses explain possible interactions between microbes and IAS. For example, the enemy release hypothesis [9, 10] suggests that the invader may leave its natural enemies (e.g. micropathogens) behind during dispersal into the novel environment, which would result in a lower prevalence of pathogenic microbes in newly established populations and/or better condition of invading individuals. Additionally, invaders may carry novel microbes that are unfamiliar to the native species in the novel environment [11, 12], which may lead to spillover of potentially pathogenic microbes to the native species, consequently giving the invaders a selective advantage in competition (spill-over or novel weapon hypothesis [13]). Furthermore, the spillback hypothesis [14] proposes that invaders may acquire microbes from the novel environment and serve as their reservoir, multiplying their (negative) impact on the native species or on the invader itself. Finally, the invader’s microbiome may have a protective role: it may interfere with the entry of micropathogens into the host’s body and prevent their establishment, growth and spread [15]. Hence, the effects of the invasion process, the characteristics of the novel environment, and the microbiome of both the invader and the novel environment may affect the species invasion success and the dynamics of range expansion.

Studies on the microbiome often focus on the bacteriome, ignoring its other components such as viruses or fungi [16–18]. The role of microbial fungi in invasion ecology so far has been under-recognized and poorly addressed, except in the case of pathogenic fungi, which cause emerging infectious diseases [19]. Some of the most damaging IAS in vulnerable freshwater ecosystems – freshwater crayfish [20] – are particularly susceptible to diseases caused by fungi and fungal-like microbes (such as oomycetes [21]), which may play a role in their invasion success [19]. Freshwater crayfish are the largest invertebrates in temperate freshwater environments, characterized by a long lifespan and an omnivorous diet consisting of benthic invertebrates, detritus, macrophytes and algae [22, 23]. They can assume the role of primary consumer, predator and/or prey in the ecosystem, which gives them the ability to integrate into the food web at many levels [23, 24], making the non-indigenous crayfish species (NICS) potent invaders of freshwater ecosystems [25]. Negative impacts of invasive NICS, such as modifying ecosystem functioning, reducing the diversity of freshwater communities, or decreasing the number of indigenous crayfish species (ICS) populations, have been documented worldwide [20, 26, 27]. However, invading a new ecosystem is an energetically demanding process that requires investing resources in population growth and expansion or, in the case of high pathogen prevalence in the novel environment, reallocating energy reserves into immunity [28, 29]. Multiple microbial fungi have been recorded in freshwater crayfish species (both NICS and ICS) as their pathogens or symbionts in general (cf. [21, 30, 31]). The study by [31] has shown that fungi are the most studied group of microbes in the context of crayfish diseases, which sheds light on the important role that fungi play as crayfish pathogens and symbionts (cf. [30]). This, together with the fact that fungi are ubiquitous in freshwater ecosystems [32], highlights their potential impact in shaping the population dynamics of crayfish – especially NICS invading a novel environment [19, 33].

The focus of this study was the mycobiome of the most successful crayfish invader in Europe, the North American signal crayfish *Pacifastacus leniusculus* (Dana, 1852), and its environment in a recently invaded Korana River in Croatia. We analyzed the mycobiomes of the water, sediment, crayfish exoskeleton and its tissues, and examined the differences in the mycobiome between different environments along the signal crayfish invasion range. The signal crayfish was first detected in the Korana River in 2011 [34] and has since been successfully spreading in both upstream and downstream directions [35, 36]. A previous study of this population’s microbiome [37] found significant differences in the bacteriome of different tissues of signal crayfish individuals along its invasion range in the Korana River. Here, we investigated whether similar differences occur in its mycobiome. To date, most crayfish-associated fungi have been detected during health monitoring surveys of crayfish individuals, or by screening for their presence in crayfish [31]. This study is the first to analyze the mycobiome of signal crayfish using internal transcribed spacer (ITS) amplicon sequencing approach. We hypothesize that the mycobiome of the signal crayfish may be shaped by different environmental conditions along the invasion range during dispersal. We aim to examine the diversity and potential differences between crayfish and environmental mycobiomes, and to explore whether local environmental conditions (i.e. differences between upstream and downstream river segments) affect the crayfish mycobiome. The results of this study may help further understanding of how the mycobiome of crayfish changes during the species’ dispersal through the novel environment.

## Methods

### Study area

Fieldwork was carried out in the lower reaches of the Korana River, located in continental Croatia, where signal crayfish is spreading in both upstream and downstream directions [36]. Korana is a 134 km long karst river belonging to the Sava catchment, with numerous natural and man-made cascades along the entire length of its watercourse [35]. The study area included 33 km of the Korana lower watercourse, covering the whole length of signal crayfish invasion range in this river. The upstream section of the studied river segment passes through a sparsely populated rural region, while the downstream section flows through the industrial zone of the city of Karlovac. Additionally, differences in water temperature were recorded along this part of the watercourse: in a previous study by [38], the water temperature at the upstream river segment was 5.6 °C lower than at the downstream segment. Apart from the different environmental conditions, the study area also includes sites that differ in crayfish community composition: dense intraspecific populations of signal crayfish (located in the center of the studied area, U2 and D1; Table 1), and less abundant heterospecific populations of signal crayfish and narrow-clawed crayfish (located at the edges of the studied area; U1 and D2; Table 1) [36].

### Sampling procedure

Crayfish sampling was conducted in the early autumn of 2018, during the period of increased crayfish activity of both sexes (i.e. before the mating season [39]). The signal crayfish individuals were captured at four sites along the above-mentioned 33 km of the Korana lower watercourse, with two sites previously categorized as upstream (U1 and U2), and the other two sites belonging to the downstream river segment (D1 and D2; Table 1) [36]. Crayfish individuals were captured using baited LiNi traps [40] which were left in the water overnight, and identified to species level by visual inspection upon capture. Captured native narrow-clawed crayfish were returned to the river and were not included in this study. A total of 110 signal crayfish individuals of both sexes were caught (Table 1), and each animal was placed in an individual container on ice and taken to the laboratory for tissue sampling. In addition to crayfish, environmental samples (water and sediment) were collected at all four sites. Water sampling was performed using sterile 1000 mL bottles, while sediment was taken as a composite sample (4–5 samples at each of the four sites which were collected approximately 1–2 m apart, from the surface of the sediment: 0–5 cm) using a sterile sampling spoon and immediately transported to the laboratory on ice.

**Table 1:** Number of collected crayfish individuals and geographical coordinates of sampling sites along the invasion range of the signal crayfish in the Korana River in 2018

Site	Number of captured crayfish individuals	X (WGS84)	Y (WGS84)
upstream 1 (U1)	27	45.320915	15.518373
upstream 2 (U2)	30	45.371918	15.521505
downstream 1 (D1)	30	45.411808	15.609231
downstream 2 (D2)	23	45.451355	15.567030

In the laboratory, the collected water samples were vacuum-filtered through 0.22 μm pore-size mixed cellulose ester (MCE) membrane filters, which were then stored at − 20 °C along with the collected sediment samples until DNA extraction. Four types of crayfish samples were collected from each individual crayfish: exoskeletal biofilm, hemolymph, hepatopancreas, and intestine (i.e. midgut and hindgut). The exoskeletal biofilm was sampled by taking cuticle swabs as described by [41]. In brief, after manual removal of loosely adhering debris (e.g. vegetation, mud or sediment) from the crayfish, the individuals were thoroughly scrubbed with a sterile brush moistened with the 0.1% NaCl, 0.15 M Tween 20 solution. The suspension was centrifuged at 10 000×g for 15 min at 4 °C, the supernatant was discarded, and the pellet of epibiotic cells was frozen at − 20 °C. Further, using a sterile needle as described by [42], we collected 400 µL of hemolymph in 200 µL of anticoagulant solution (0.49 M NaCl, 30 mM trisodium citrate, 10 mM EDTA) from the base of the individual’s walking leg, previously rinsed by 70% ethanol. The collected hemolymph was centrifuged at 10 000×g for 10 min at 4 °C, the supernatant was discarded, and the pellet was frozen at − 20 °C until DNA extraction. After hemolymph sampling, each individual was killed according to available guidelines for the humane killing of crayfish (rapid cut of the nerve cord from the thorax to the end of the abdomen [43]). Each animal was then dissected, and the same sampling procedure was used to obtain both the hepatopancreas and the intestine: the complete organ was removed from the body, placed in a sterile Petri dish and carefully chopped into small pieces using a sterile scalpel, and frozen at − 20 °C. Non-disposable dissecting equipment was alcohol flame sterilized between each individual sample.

### DNA extraction

Genomic DNA was extracted from four types of crayfish samples (exoskeletal biofilm, hemolymph, hepatopancreas and intestine) using the NucleoSpin Microbial DNA kit (Macherey-Nagel, Germany) according to the manufacturer’s protocol, with modifications regarding sample lysis by agitation as described by [41]. Genomic DNA from sediment and water samples was extracted using the DNeasy PowerSoil Pro Kit (Qiagen, Germany). A total of three replicates of each composite sediment sample were isolated from the upstream river segment samples, and three from the downstream river segment samples. DNA quantity was analyzed in all samples using the QuantiFluor ONE dsDNA System and the Quantus Fluorometer (Promega, USA). Finally, based on satisfactory DNA concentration, we selected 192 samples from all six sample groups for amplicon sequencing of the variable region ITS2 of the ITS region between genes encoding for small and large subunits of fungal ribosomal RNA (Additional Table 1 A).

### Library preparation, sequencing and bioinformatic analysis

Amplification and sequencing of the variable ITS2 region was performed by Microsynth, Switzerland. The Illumina library was prepared using ITS Nextera two-step PCR using forward ITS3 (5′- GCATCGATGAAGAACGCAGC − 3′) and reverse ITS4 (5′- TCCTCCGCTTATTGATATGC − 3′) primers [44], and sequenced on an Illumina MiSeq using the MiSeq Reagent Kit v2 (2 × 250 bp paired-end). The analyses of Illumina raw paired-end sequences were conducted using the ‘Quantitative Insights Into Microbial Ecology 2’ (QIIME2) software [45], release 2021.2. After importing raw demultiplexed paired-end fastq files into QIIME2 using a manifest file, they were quality filtered, trimmed, dereplicated, denoised, merged and assessed for chimeras to produce amplicon sequence variants (ASVs) using the DADA2 plugin [46]. The DADA2 generated feature table was filtered to remove singletons. Taxonomy was assigned to ASVs using a pre-trained Naïve Bayes classifier. The classifier was trained on the UNITE version 8.3 database of reference sequences clustered at 99% sequence similarity [47] using the QIIME2 feature-classifier plugin [48]. Based on the generated taxonomy, the feature table was filtered to exclude all ASVs assigned to kingdoms other than Fungi. After obtaining the final feature table, hemolymph and hepatopancreas samples were excluded from further analyses, due to the low number of reads obtained per sample (median frequency per sample: 112.5 for hepatopancreas, 138 for hemolymph, 33 120 for exoskeleton, 604 for intestine, 10 319 for water, 4 523 for sediment). In addition, large taxonomical differences in the observed ASVs were found in hemolymph and hepatopancreas samples from different crayfish individuals (data not shown), and since we could not distinguish between biologically relevant data and possible artifacts introduced in the amplification step, we have decided not to present these data altogether. A phylogenetic tree was constructed using fasttree2 based on mafft alignment of the ASVs as implemented in the q2-phylogeny plugin. The mycobiome diversity and richness of all sample types (four sample groups: water, sediment, exoskeletal biofilm and intestine) were compared using alpha (observed features and Pielou’s evenness index) and beta (Jaccard index and Bray-Curtis dissimilarity) diversity metrics using the diversity plugin within QIIME2. For these analyses, the samples were subsampled to a minimum of 1 064 reads per sample. This threshold was chosen to avoid excessive loss of samples (leaving a total of 69 samples for the diversity analyses, Additional Table 1B), even though the generated rarefaction curve was not saturated (Additional Fig. 1). Additionally, due to the bidirectional spread of the signal crayfish in the Korana River, we analyzed the differences in composition of mycobiome at upstream (U1 and U2) and downstream (D1 and D2) sites along its range, since we presume that these rivers sections differ in their environmental conditions (i.e. water temperature, anthropogenic pressure; described in the section Study area, Methods). For these analyses, sample groups were subsampled to 4 717 reads per sample for water, 1 603 for sediment, 1 201 for exoskeletal biofilm, and 1 064 for intestine. Since no significant differences between sexes were recorded for any of the crayfish sample groups (exoskeletal biofilm, intestine) in both alpha and beta diversity analyses, the sexes were pooled together. Differences between all four sample groups and between upstream and downstream river segments were tested with Benjamini–Hochberg corrected Kruskal–Wallis and permutational multivariate analysis of variance (PERMANOVA) tests [49] for alpha and beta diversity, respectively. Visualization of beta diversity metrics was made by generating principal coordinates analysis (PCoA) plot using Emperor [50]. Shared and unique ASVs between all four sample groups were determined using Venn diagrams, visualized with an online tool (http://bioinformatics.psb.ugent.be/webtools/Venn/). Additionally, analysis of compositions of microbiomes (ANCOM) tests [51] were used to identify ASVs that differ in abundance between upstream and downstream river segments using the composition plugin within QIIME2.

## Results

After processing the reads using the DADA2 plugin [46] and filtering of the resulting feature table (i.e. removing singletons, eukaryotes other than fungi, hemolymph and hepatopancreas samples), 2 306 526 merged reads from 112 samples were obtained, and a total of 7 461 ASVs were identified. The relatively low number of ASVs which was obtained in hemolymph and hepatopancreas samples points to a small fungal abundance in these tissues.

### Diversity and composition of environmental and crayfish mycobiomes

#### Alpha and beta diversity

**Fig. 1:**
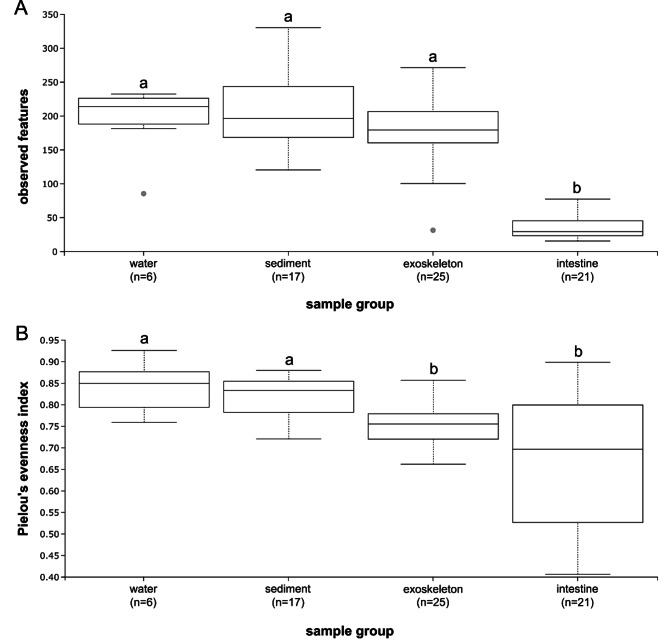
Alpha diversity analyses showing (A) the number of observed features and (B) the Pielou’s evenness index for mycobiomes from different sample groups. Significant differences are marked with different letters

Overall, community richness, presented as the number of observed features, differed significantly (Kruskal-Wallis test: P = 2.60E–09, H = 42.89) between all samples. However, pairwise comparisons showed that crayfish intestine samples had a significantly lower number of observed fungal features (i.e. lower richness) compared to the other three sample groups (Fig. 1A). No significant differences in the number of observed features were recorded between exoskeleton, sediment and water (Fig. 1A). Furthermore, the overall fungal community evenness, presented as Pielou’s evenness index, also differed significantly (Kruskal-Wallis test: P = 6.09E–04, H = 17.31) between all samples. Pairwise comparisons showed significantly higher evenness in water and sediment compared to exoskeleton and intestine (Fig. 1B). No significant differences in evenness were recorded between sediment and water, and exoskeleton and intestine (Fig. 1B).

**Fig. 2:**
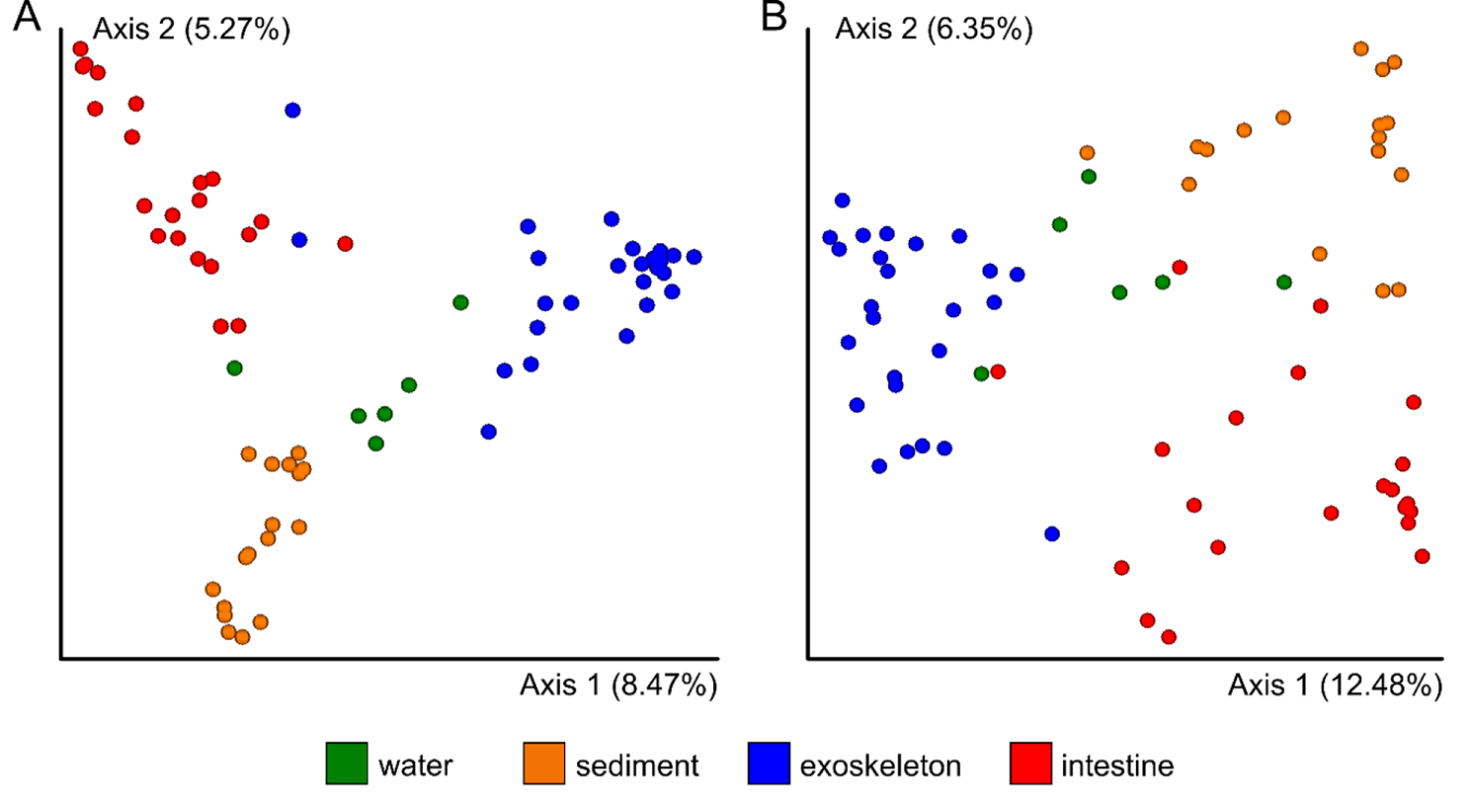
Beta diversity analyses of mycobiomes between different sample groups. The PcoAs are based on (A) Jaccard and (B) Bray-Curtis distance matrices

Both beta diversity analyses (i.e. Jaccard index and Bray-Curtis dissimilarity) showed overall significant differences (PERMANOVA test: P = 0.001, pseudo-F = 3.77 and P = 0.001, pseudo-F = 5.01, respectively) between mycobiomes of all four sample groups. Further, beta diversity pairwise tests exhibited significant differences between all pairs of sample groups (P = 0.001). PCoAs based on Jaccard and Bray-Curtis distance matrices show a clear separation of all four sample groups (Fig. 2).

#### Mycobiome composition, shared and unique ASVs

**Fig. 3:**
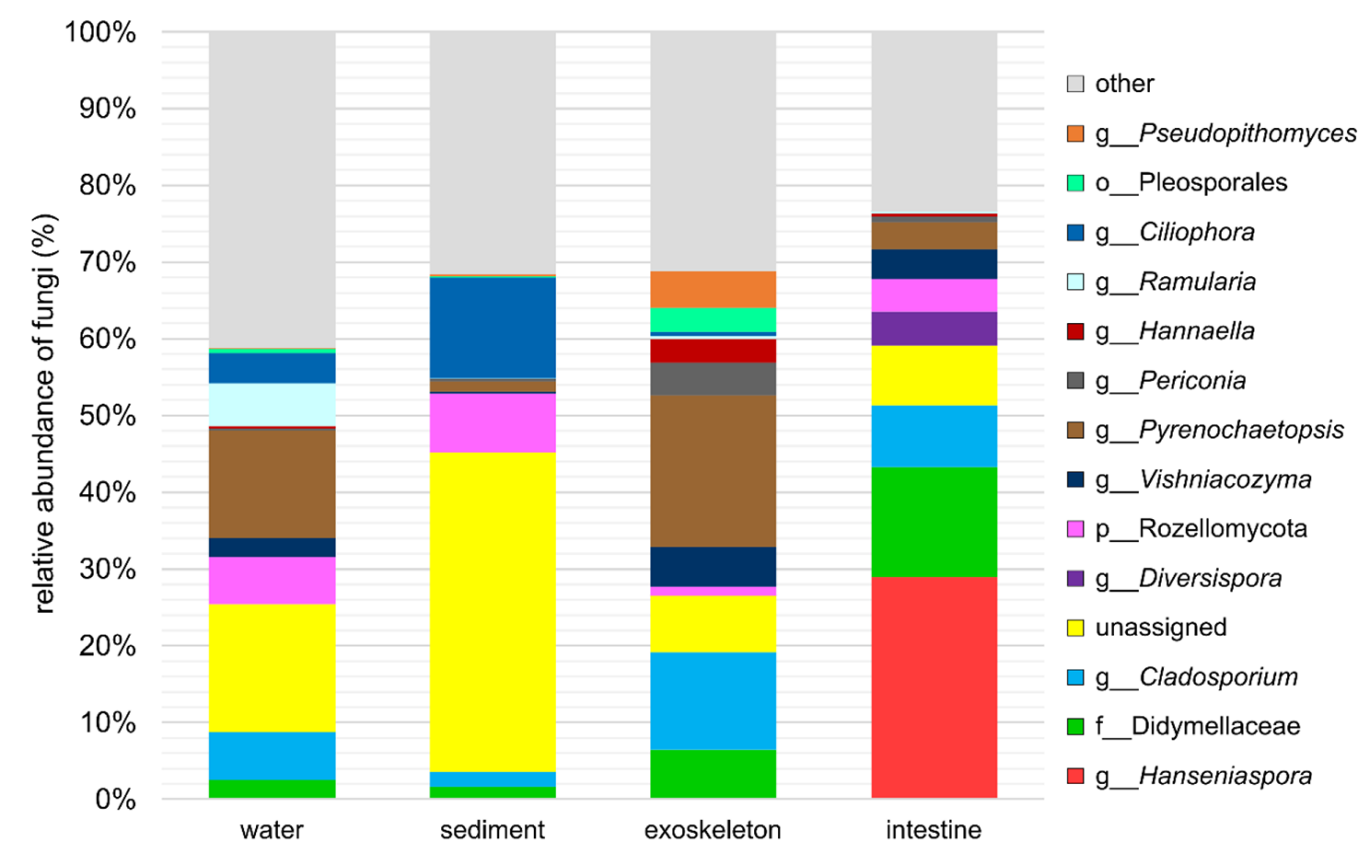
Relative abundance (%) of the overall most prevalent genera of all four sample groups. ASVs that could not be identified to genus (g) level are marked with the letter corresponding to the last known taxonomic level (p = phylum, o = order, f = family). Fungal taxa with a total abundance of > 3% are shown, while the remaining taxa were pooled and marked as “other.” ASVs to which taxonomy could not be assigned were pooled and marked as „unassigned“

A total of 856 different fungal taxa were detected at the genus level in all four sample groups. The genus *Pyrenochaetopsis* (phylum Ascomycota) was the most abundant genus in water (14.00%) and exoskeletal (19.80%) mycobiomes, but it was relatively poorly represented in sediment and intestinal mycobiomes (< 4%). The genus *Ciliophora* (Ascomycota) comprised 13.13% of sediment mycobiome, but only 3.88% of water and < 1% of exoskeletal mycobiomes, and it was completely absent from crayfish intestine. Intestinal samples were dominated by the ascomycete genus *Hanseniaspora* (28.94%), which constituted only a small percentage (< 0.02%) of the mycobiomes of other three sample groups. Additionally, an undetermined genus from the family Didymellaceae (Ascomycota) showed notable abundance of 14.33% in the intestinal mycobiome, but composed a relatively small percentage (< 6.5%) of other sample groups’ mycobiomes. The category „other“, which included a total of 842 taxa with < 3% abundance, constituted between 23.42% and 41.22% of all sample groups’ mycobiomes. However, taxonomy could not be assigned for a relatively high percentage of ASVs in each sample group, especially in environmental samples (water 16.61%, sediment 41.59%, exoskeleton 7.42% and intestine 7.79%; marked as unassigned, Fig. 3).

**Fig. 4:**
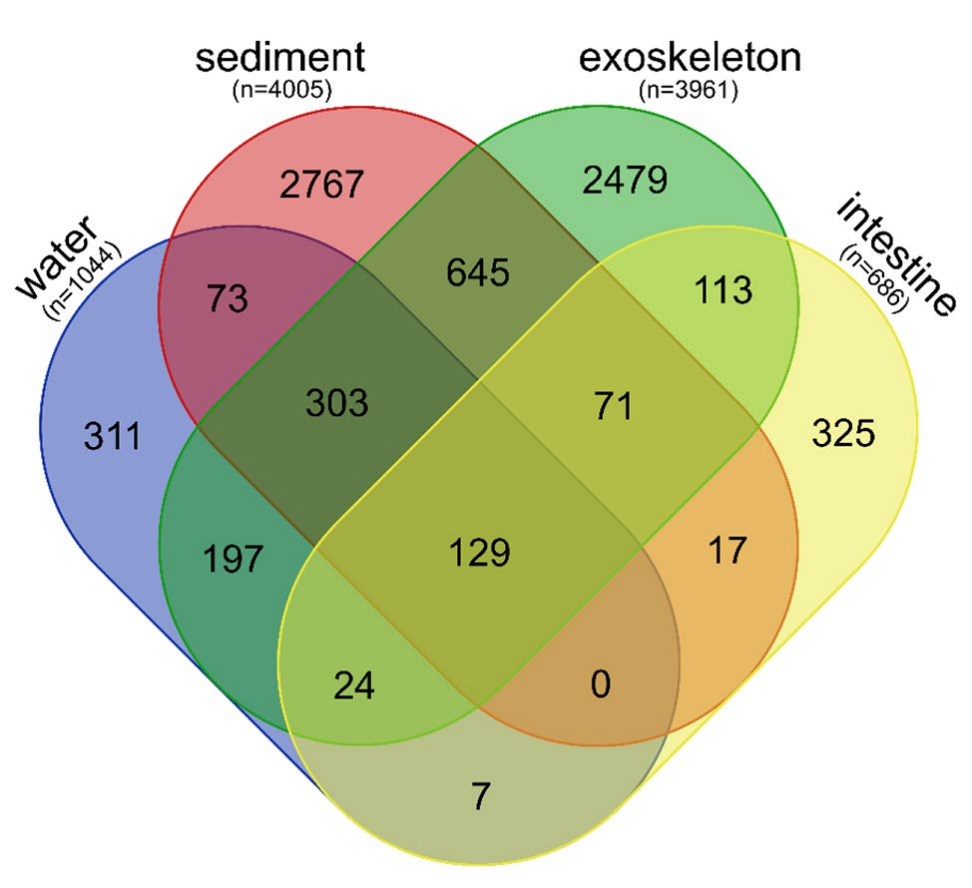
Venn diagrams showing the numbers of shared and unique ASVs between four sample groups. The total number of ASVs in each sample group is marked with n

Comparisons of shared and unique ASVs between the four sample groups showed that the exoskeleton shared the highest number of ASVs with the sediment samples (645 or 16.28% of all exoskeletal ASVs; Fig. 4). The intestine shared the least ASVs with any of the environmental samples (7 or 1.02% with water, and 17 or 2.48% with sediment), but shared 16.47% (113) of its ASVs with the exoskeleton. Environmental samples – water and sediment – shared a relatively low amount of ASVs (73, accounting for 6.99% of all water ASVs, and 1.82% of all sediment ASVs). Unique ASVs were highly represented in the sediment samples (69.09%, 2 767), followed by exoskeleton (62.59%, 2 479) and intestine (47.38%, 325), while water had the lowest percentage of unique ASVs (29.79%, 311).

### Comparison of environmental and crayfish mycobiomes between upstream and downstream river segments

**Fig. 5:**
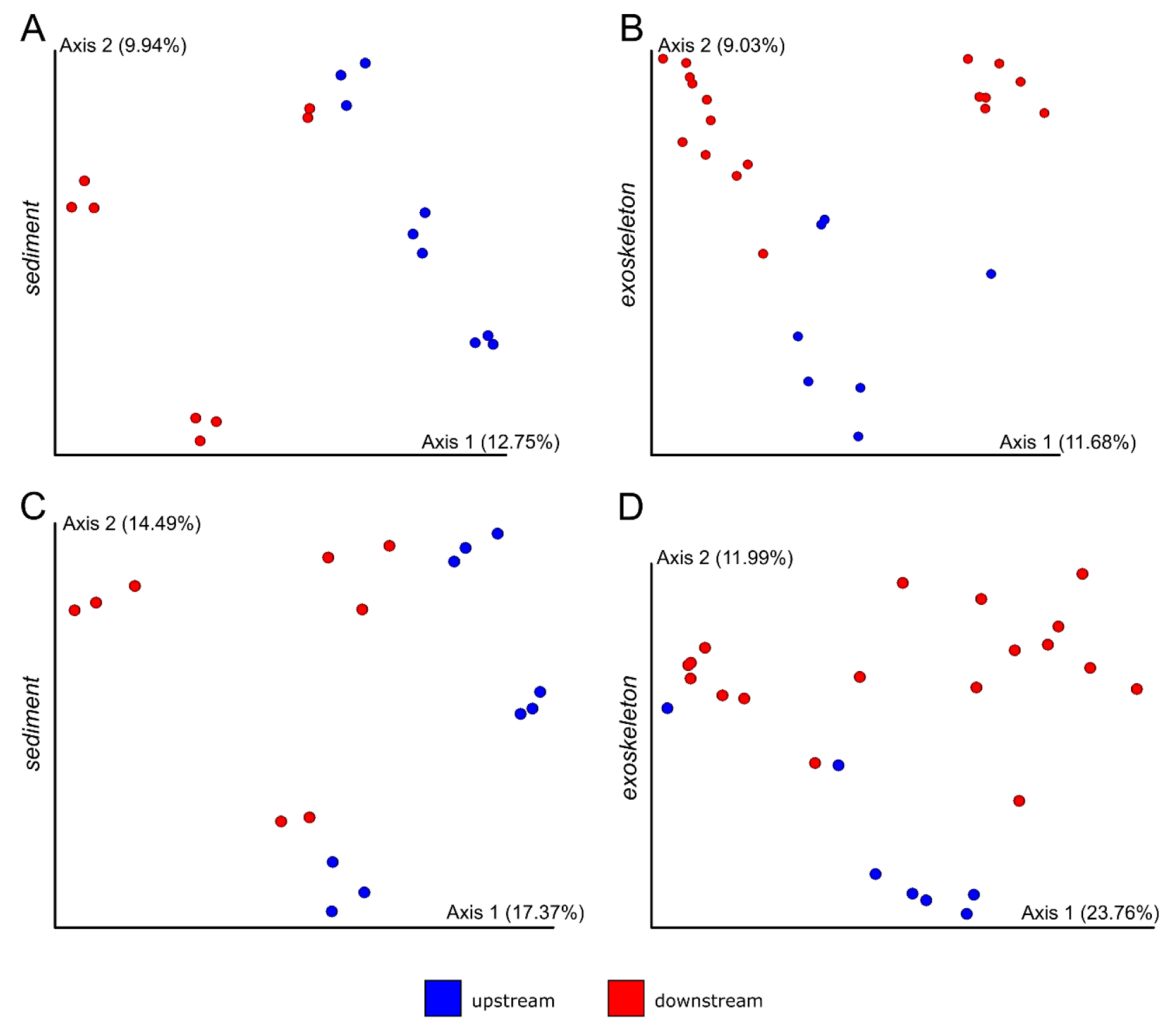
Beta diversity analyses of (A,C) sediment and (B,D) exoskeletal mycobiomes between upstream and downstream river segments. The PcoAs are based on (A,B) Jaccard and (C,D) Bray-Curtis distance matrices

Alpha and beta diversity analyses were used to examine potential differences between mycobiomes of upstream and downstream river segments in all four sample groups. No significant differences in alpha diversity (i.e. richness) between two river segments were found for any of the four sample groups. However, significant differences between upstream and downstream mycobiomes were recorded for sediment (Fig. 5A&C) and exoskeleton (Fig. 5B&D) in both beta diversity analyses used: Jaccard index (PERMANOVA test: P = 0.002, pseudo-F = 1.94 for sediment and P = 0.001, pseudo-F = 1.99 for exoskeleton; Fig. 5A&B) and Bray-Curtis dissimilarity (PERMANOVA test: P = 0.001, pseudo-F = 2.39 for sediment and P = 0.007, pseudo-F = 2.36 for exoskeleton; Fig. 5C&D). Finally, beta diversity analyses showed no significant differences between upstream and downstream river segments in the case of water and intestinal mycobiomes.

Differential abundance analysis (i.e. ANCOM) performed at the genus level detected two ASVs of significantly different abundance between upstream and downstream river segments in the exoskeletal mycobiome: the genera *Ascochyta* (W-statistic value = 541, clr mean difference = 3.045) and *Leptosphaeria* (W-statistic value = 536, clr mean difference = 2.378). No significant differences in ASV abundance between the two river environments were recorded for water, sediment and intestine mycobiomes.

## Discussion

This study provides the first insight into the composition and diversity of the signal crayfish mycobiome, and into mycobiome changes during range expansion due to environmental effects on the IAS population. In this study, the signal crayfish mycobiome was analyzed for the first time using data based on amplicon sequencing of the ITS region of the rRNA gene. Due to overall lack of research of crayfish mycobiome, and the absence of cultivation-independent data based on high throughput sequencing, only limited comparative information was available in the literature, except for several studies that analyzed fungal isolates from crayfish by sequencing the ITS region (e.g. [52, 53]).

### Diversity and composition of environmental and crayfish mycobiomes

Of all the tissues sampled (exoskeleton, hemolymph, hepatopancreas, intestine), the diversity and composition of the mycobiome was successfully analyzed only for the exoskeleton and intestine: upon obtaining the results of sequencing, hemolymph and hepatopancreas samples were excluded from the downstream analyses due to a low number of reads (in contrast to other sample groups where ITS was successfully amplified, discussed later in the text).

Among the samples that were included in the downstream analyses, the highest richness and evenness were recorded in fungal communities of environmental samples (water and sediment), compared to the crayfish samples (exoskeleton and intestine). As richness and evenness are the main components of biodiversity [54, 55], our results indicate, expectedly, that the environmental mycobiomes have higher diversity than the crayfish-associated mycobiomes. Furthermore, the intestine exhibited the lowest number of observed ASVs (i.e. the lowest richness of the mycobiome) in comparison to the exoskeleton, water and sediment. This low mycobiome richness can be explained by the fact that fungi which enter the intestine via the available food sources are filtered through several selective processes. On their way through the digestive tract, they are exposed to the digestive enzymes of the hepatopancreas and chemically broken down even before entering the intestine [56]. Also, due to the homeostasis of crayfish organism and the existing intestinal microbiome, the intestine (and internal tissues in general) may be more resistant to mycobiome changes in comparison to other sample groups, that are either a part of the environment (water, sediment) or in constant contact with the environment (exoskeleton), and therefore may contain higher fungal diversity due to dynamic and constantly changing conditions. Additionally, the intestine may serve as a primary niche for certain fungal taxa, as reported in mammals [57]. For example, in this study, crayfish intestine exhibited a high abundance of the yeast genus *Hanseniaspora*, while the same genus was present in very low abundance in water, sediment and exoskeleton. This indicates that either crayfish consume food rich in this yeast, or that this yeast is an integral part of their intestinal mycobiome. Furthermore, the genus *Diversispora* was recorded only in crayfish intestine and was absent from other sample groups. Members of this genus are fungal symbionts of vascular plants [58], hence their presence in crayfish intestine may be explained by the crayfish diet (i.e. grazing), since the intestinal microbiome reflects the feeding style of the host [59]. On the contrary, the genus *Ciliophora* was present in all sample groups except in the intestine. The genus *Ciliophora* contains two known species (*C. cryptica* and *C. quercus*), both of which are endophytic fungi [60]. Therefore, due to symbiosis with plants, this genus is present in the crayfish environment and can be found attached to crayfish exoskeleton. However, our results indicate that the plants which are hosts of this genus do not constitute a significant part of the crayfish diet.

Beta diversity analyses showed that all four analyzed sample groups had distinct mycobiome composition and ASV abundance, suggesting that their mycobiomes are shaped by different factors (e.g. molting frequency, diet), and/or are inhabited by fungal species that prefer a certain tissue or environment as a niche. This is not surprising, since the four analyzed sample groups differ in physical and chemical properties, and therefore provide different microhabitats for colonizing microbes, including fungi (cf. [59, 61, 62]). A similar uniqueness was also found for the bacteriome of the signal crayfish and its environment [37].

Comparisons of shared and unique ASVs between four sample groups showed that the exoskeletal and sediment mycobiomes shared the highest number of ASVs, indicating that exoskeletal mycobiome is at least partly shaped by the sediment mycobiome (discussed later in the text). Further, intestinal mycobiome shared more ASVs with the exoskeleton than with the environmental samples, suggesting that the fungi shared between these two sample groups may be crayfish symbionts. The highest number of unique ASVs was recorded in the sediment and exoskeletal samples. This is not surprising, as both sediment and exoskeleton are highly diverse sample groups – before filtering, these sample groups contained taxa from 10 eukaryotic kingdoms other than Fungi (data not shown).

As mentioned above, a low number of reads was obtained from hemolymph and hepatopancreas samples. Such result could reflect the low abundance and diversity of fungi in these tissues. Although crustacean hemolymph is not sterile [63] and may harbor bacteria [31, 37], microbial fungi may be poorly represented in this tissue potentially due to immune defense system (prophenoloxidaze-activating system) which is activated in contact with microbial products such as β-1,3-glucans (present in the cell walls of fungi [64]), which results in their removal. On the other hand, the hepatopancreas is a digestive gland that plays an important role in food degradation, and hence has a specific organ environment (i.e. low pH, presence of digestive enzymes [65]), that may impede the colonization of this organ by microbial fungi. Also, due to the biology of microbial fungi and their specific life-cycle characteristics (i.e. their hyphae penetrating the cuticle and growing inside the exoskeleton and the tissues immediately beneath), they can mostly be found on the surface of crustacean exoskeleton [31, 66]. However, in the case of severe infection, microbial fungi can be found in other tissues, including hemolymph [67] and hepatopancreas [68].

In this study, we have decided not to present the data on ITS reads obtained from hemolymph and hepatopancreas samples because of the insufficient number of reads, and large differences in the observed features in samples originating from different crayfish individuals. Presenting these data would lead to uncertainty in the interpretation of the results and the inability to distinguish between biologically relevant data and possible artifacts introduced in the amplification step. In the future, the quantification of fungi in different crayfish tissues should be performed (e.g. by qPCR) to confirm the presumption that fungi are present in low abundance in the hemolymph and hepatopancreas, compared to the exoskeleton and intestine. Also, metagenomic analyses of RNA datasets, that do not involve the bias-prone PCR-amplification step applied in this study, could be useful to gain insight into the viable fraction of rare fungal taxa inhabiting these crayfish tissues.

### Comparison of environmental and crayfish mycobiomes between upstream and downstream river segments

Sediment and exoskeletal mycobiomes differed significantly between upstream and downstream river segments in both composition and ASV abundance. As previously described in the Methods section, these two river segments differ in local environmental conditions and water temperature. Multiple studies have confirmed that conditions of the local environment and water parameters can affect and shape the microbiome of the host’s body surface (e.g. in fish: [69–71]). Furthermore, the sediment microbiome can also be affected by numerous external factors, such as the presence of macrophyte roots, the amount of nutrients and organic matter, metal concentrations, pollution, and the type of land use [72–75]. Finally, co-occurring (benthic) animal species and their abundance in different river segments may affect both exoskeletal microbiome (by sharing microbes during frequent social and agonistic interactions, or during mating [76, 77]) and sediment microbiome (through bioturbation, or feeding on detritus [78, 79]).

Apart from the above-mentioned external factors that may affect the mycobiomes of exoskeleton and sediment along the upstream and downstream river segments, these two mycobiomes may also shape each other. Since crayfish are benthic organisms and bioturbators, their exoskeleton is continuously in close contact with the sediment, and simultaneously acts as a barrier and a link between crayfish and their environment [80, 81]. Crayfish movement through sediment, and its following disturbance, may ultimately enable/aid the colonization of the crayfish exoskeleton by (novel) microbial fungi from the sediment. This is supported by our results, showing that the sediment and the exoskeleton are the two sample groups that share the highest number of ASVs. Furthermore, previous research of crayfish microbiome [37, 82] showed that the exoskeletal bacteriome is partly shaped by the sediment bacteriome, which may also be possible in the case of the mycobiome. Therefore, changes in the sediment mycobiome (triggered by, for example, a change in the local environmental conditions) may eventually lead to changes in the exoskeletal mycobiome. However, a large number of unique ASVs, which was recorded in both sediment and exoskeleton, indicates that the exoskeletal mycobiome is also partly shaped by factors other than the environment (i.e. sediment) which is consistent with the conclusions of previous exoskeletal microbiome research [37]. In addition, [62] report that the process of molting and subsequent formation of a new exoskeleton may affect the microbiome of multiple tissues in mud crabs. Therefore, molting is an important factor that should be taken into consideration when researching exoskeletal microbiome (but also microbiomes of other tissues of ectodermal origin) in crustaceans, especially in juvenile individuals that molt more frequently.

Differential abundance analysis showed that two fungal genera inhabiting the crayfish exoskeleton (*Ascochyta* and *Leptosphaeria*) were more abundant in the individuals at downstream river segment in comparison to upstream. Both of these genera mainly include species that are plant pathogens [83, 84]. Further, some members of the genus *Ascochyta* produce brefeldin A (which showed antifungal, antiviral, and anticancer activity [85]), while some members of the genus *Leptosphaeria* produce bacteriocins (which showed inhibitory and antimicrobial activity against some bacteria [86]). These fungal products, which have antibacterial, antiviral and/or antifungal properties, may also shape the exoskeletal microbiome of crayfish by inhibiting its colonization by potential pathogens. Similarly, a study by [87] has demonstrated that some bacteria present on the crayfish exoskeleton can inhibit the growth of the crayfish fungal-like pathogen *Aphanomyces astaci* Schikora, 1906. Further studies are needed to elucidate the role of antimicrobial fungal products in the crayfish exoskeleton, and to examine whether the higher abundance of genera *Ascochyta* and *Leptosphaeria* in individuals at downstream river segment has any effect on the health status or pathogen load of the crayfish at downstream river segment, in comparison to upstream.

## Conclusions

This study has shown that the mycobiome of signal crayfish exoskeleton significantly differs along the invasion range and exhibits similar pattern of change as the sediment mycobiome, indicating that different local environmental conditions may shape the exoskeletal mycobiome during range expansion. Since intestinal mycobiome did not differ significantly between different local environmental conditions, we suggest that it may be more conserved and resistant to change, and/or is mainly shaped by the more uniform crayfish dietary preferences (although crayfish are omnivores, adult individuals generally have a plant-based diet, whereas juveniles have an animal-based diet, and also exhibit seasonal changes in feeding habits [88, 89]). Crayfish-associated mycobiomes and their effects on crayfish hosts are still largely unexplored. Therefore, future studies are needed in order to determine how the mycobiome and its changes affect crayfish individuals and population dynamics, especially in the case of NICS. Our study provides a foundation for future research on crayfish-associated fungi, required to assess the contribution of the mycobiome to an individual’s overall health status and resistance to pathogens and diseases, and ultimately, to assess its contribution to invasion success.

## Electronic supplementary material

Below is the link to the electronic supplementary material.

Supplementary Material 1

## Data Availability

The datasets generated and/or analyzed during the current study are available in the EMBL Nucleotide Sequence Data Base (ENA) repository, https://www.ebi.ac.uk/ena/browser/home, reference number PRJEB54977.
