# Supplementary material for: The mycobiome of a successful crayfish invader and its changes along the environmental gradient
Source: Anim Microbiome. 2023 Apr 11;5:23. doi: 10.1186/s42523-023-00245-9 (PMC10088235; doi:10.1186/s42523-023-00245-9)

**ADDITIONAL FILES**

**ADDITIONAL TABLE 1.** Number of sequenced and analyzed samples per site and sample type.

| A | samples chosen for amplicon sequencing of variable region ITS2 of the ITS region of fungal ribosomal DNA (192 samples) | | | | | |
| --- | --- | --- | --- | --- | --- | --- |
|  | **water** | **sediment** | **exoskeletal biofilm** | **hemolymph** | **hepatopancreas** | **intestine** |
| upstream 1 | 2 | 6 | 8 | 12 | 10 | 14 |
| upstream 2 | 1 | 3 | 8 | 11 | 10 | 12 |
| downstream 1 | 1 | 3 | 12 | 11 | 12 | 13 |
| downstream 2 | 2 | 6 | 11 | 7 | 6 | 11 |
| TOTAL | 6 | 18 | 39 | 41 | 38 | 50 |
| B | **samples included in alpha and beta diversity analyses (69 samples)** | | | | | |
|  | **water** | **sediment** | **exoskeletal biofilm** | **hemolymph** | **hepatopancreas** | **intestine** |
| upstream 1 | 2 | 6 | 5 | excluded from these analyses | excluded from these analyses | 8 |
| upstream 2 | 1 | 3 | 2 |  |  | 2 |
| downstream 1 | 1 | 2 | 11 |  |  | 7 |
| downstream 2 | 2 | 6 | 7 |  |  | 4 |
| TOTAL | 6 | 17 | 25 | 0 | 0 | 21 |

**ADDITIONAL FIGURE 1.** Alpha rarefaction plot of observed features against sampling depth for each sample group.


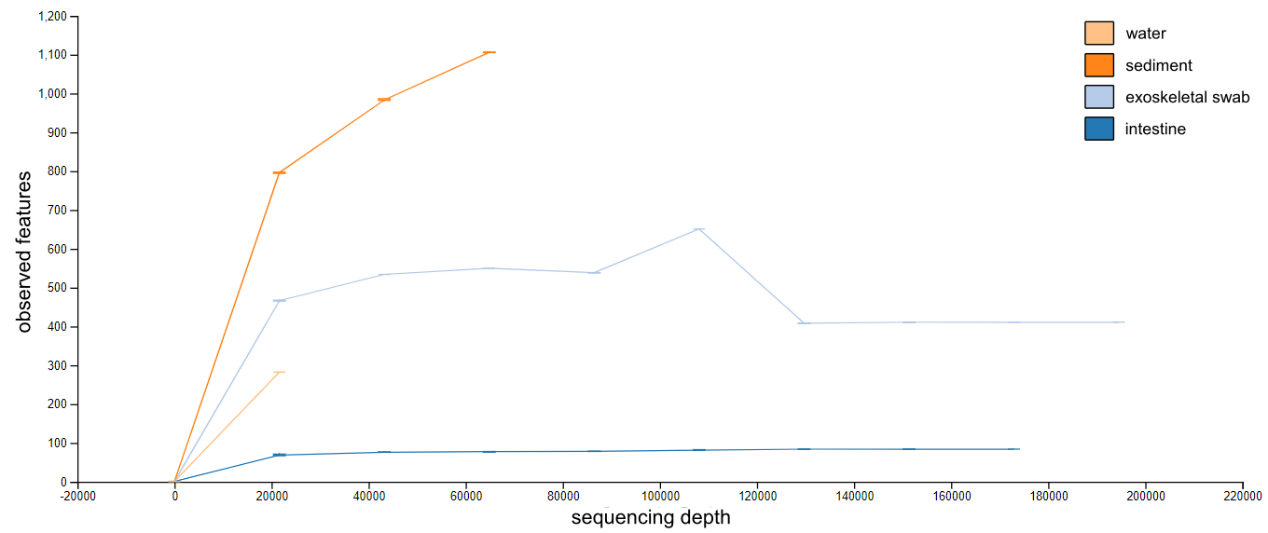

Supplement: Supplementary file 1 — Supplementary Material 1 [file 42523_2023_245_MOESM1_ESM.docx]
